# Circumferential SH Wave Piezoelectric Transducer System for Monitoring Corrosion-Like Defect in Large-Diameter Pipes

**DOI:** 10.3390/s20020460

**Published:** 2020-01-14

**Authors:** Hao Zhang, Yuehao Du, Jihua Tang, Guozheng Kang, Hongchen Miao

**Affiliations:** Applied Mechanics and Structure Safety Key Laboratory of Sichuan Province, School of Mechanics and Engineering, Southwest Jiaotong University, Chengdu 610031, China

**Keywords:** SH guided waves, circumferential guided waves, mode conversion, wall thinning defects, reflection and transmission coefficients

## Abstract

The fundamental circumferential shear horizontal (CSH_0_) wave is of practical importance in monitoring corrosion defects in large-diameter pipes due to its virtually non-dispersive characteristics. However, so far, there have been limited CSH_0_ wave transducers which can be used to constitute a structural health monitoring (SHM) system for pipes. Moreover, the CSH_0_ wave’s capability of sizing the corrosion-like defect has not yet been confirmed by experiments. In this work, firstly, the mechanism of exciting CSH waves was analyzed. A method based on our previously developed bidirectional SH wave piezoelectric transducers was then proposed to excite the pure CSH_0_ mode and first order circumferential shear horizontal (CSH_1_) mode. Both finite element simulations and experiments show that the bidirectional transducer is capable of exciting pure CSH_0_ mode traveling in both circumferential directions of a 1-mm thick steel pipe from 100 to 300 kHz. Moreover, this transducer can also serve a sensor to detect CSH_0_ mode only by filtering circumferential Lamb waves over a wide frequency range from 100 to 450 kHz. After that, a method of sizing a rectangular notch defect by using CSH_0_ wave was proposed. Experiments on an 11-mm thick steel pipe show that the depth and circumferential extent of a notch can be accurately determined by using the proposed method. Finally, experiments were performed to investigate the reflection and transmission characteristics of CSH_0_ and CSH_1_ waves from notches with different depths. It was found that transmission coefficients of CSH_0_ mode decrease with the increasing of notch depth, which indicates that it is possible to monitor the depth change of corrosion defects by using CSH_0_ wave.

## 1. Introduction 

Wall thinning due to corrosion in pipes is a serious problem in the oil and chemical industries. The ultrasonic bulk-wave-based nondestructive testing (NDT) technique and magnetic flux leakage method are available for detecting wall thinning. However, these NDT techniques cannot monitor the growth of damage, since they are usually conducted at regularly scheduled intervals. The limitation of NDT techniques motivates the development of structural health monitoring (SHM) techniques, with the aim of monitoring the overall integrity of structures in a real-time manner [1].

**Figure 1:**
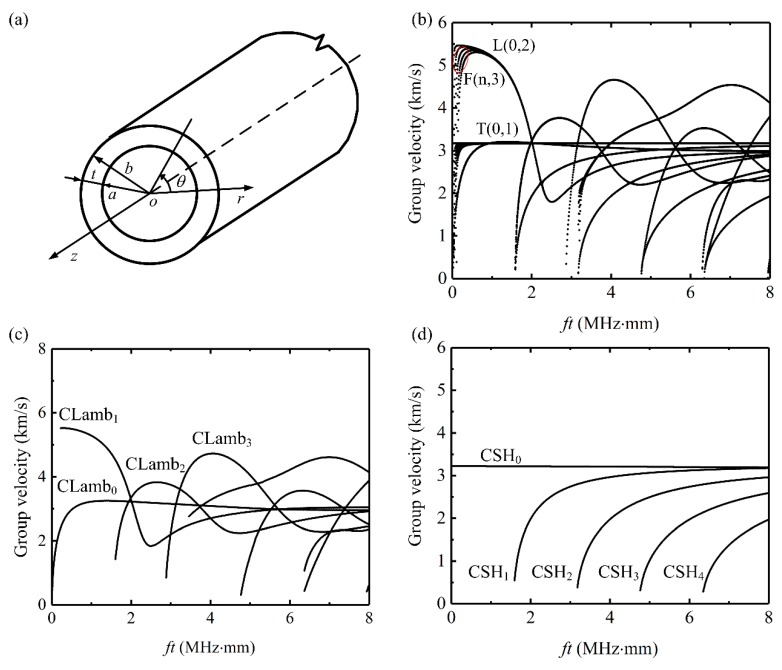
Group velocity dispersion curves of axial and circumferential guided waves in a steel pipe (outer diameter 720 mm, wall thicknesses 11 mm, Young’s modulus 210 GPa, density 7850 kg/m^3^ and Poisson’s ratio 0.33): (**a**) pipe axes configuration; (**b**) axial guided waves (F modes only shown up to 4th order); (**c**) circumferential Lamb (CLamb) waves and; (**d**) circumferential shear horizontal (CSH) waves.

Over the last three decades, the ultrasonic guided-wave-based damage identification approach has demonstrated potential for SHM of pipes [2]. Guided waves can propagate in the axial or circumferential direction of a pipe-like structure [3]. Axial guided waves have attracted the main research interest of this field due to their rapid defect screening capability. Figure 1b shows the group velocity dispersion curves of axial guided waves for a steel pipe (outer diameter 720 mm, wall thicknesses 11 mm). Based on the early works by Gazis [4,5] and Silk and Bainton [6], there are three kinds of axial guided waves in a pipe, namely longitudinal-type modes L(0, m), torsional-type modes T(0, m) and flexural modes F (n, m), where the positive integer ‘n’ represents the circumferential order of a mode and the positive integer ‘m’ denotes the group order of a mode. Since many guided wave modes exist in the axial direction at any given frequency and they are in general dispersive, it is often very difficult to extract relevant information on defects from the complex received wave signals. Therefore, much effort has been made to excite pure longitudinal L(0, 2) mode [7,8,9] and the fundamental torsional wave T(0, 1) mode [10,11], since T(0, 1) wave is nondispersive at all frequencies while L(0, 2) wave is virtually non-dispersive in the selected frequency range [12]. However, the L(0, 2) and T(0, 1) modes are axially symmetric, so they have difficulty to locate the defect circumferential position and size the defect. This problem becomes more serious in large-diameter pipes, which drives researchers to develop circumferential guided-wave-based damage identification method [13,14,15,16,17].

**Figure 2:**
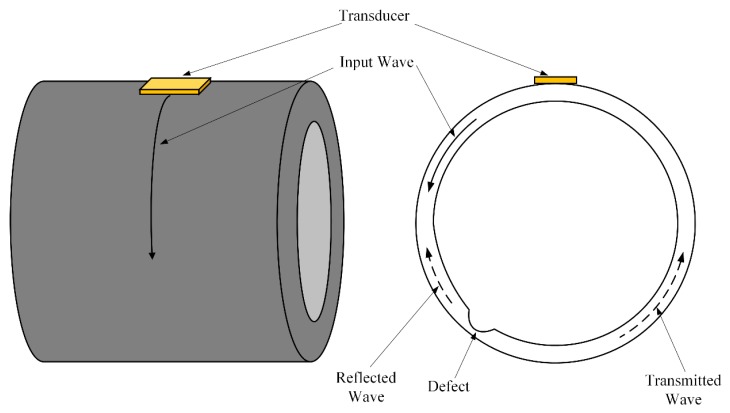
Schematic of circumferential guided-wave-based SHM approach.

Figure 2 illustrates the basic idea of the circumferential guided-wave-based SHM approach [16]. Transducers are permanently installed on the pipe. The incident guided wave is excited in one direction around the circumference of a pipe and propagates across the defect. By examining the reflected wave and transmitted wave, the defect can be identified. Since the required inspection distance is relatively shorter than that of the axial guided wave, a higher frequency circumferential guided wave can be used to improve the sensitivity to defects. Obviously, circumferential guided-wave-based inspection is an appealing method for monitoring large-diameter pipes such as pressure vessels and oil tanks, in which a transducer array can be used. As for long range pipes, such as oil transporting pipelines, circumferential guided waves can be a good supplement to axial guided wave inspection. Long range pipes can be inspected by using a combination of axial and circumferential guided waves. For example, circumferential guided waves can be used to inspect the welding zones and pipe support regions, which are highly prone to generating defects [18].

The selection of a proper mode is essential to developing a circumferential guided-wave-based SHM system. Two kinds of guided waves can propagate in the circumferential direction of a pipe, namely: circumferential Lamb waves and shear horizontal (SH) waves often abbreviated as CLamb waves and CSH waves [3], respectively, as shown in Figure 1c,d. Early researchers often use plate-wave solutions to model the circumferential guided waves. Liu and Qu [19] and Zhao and Rose [20] developed detailed theoretical models for circumferential guided waves. Their results show that the dispersion curves and wave structures of circumferential guided waves are quite different from that of plate waves especially in thick wall pipes. As shown in Figure 1c, all the circumferential Lamb waves are dispersive and at least two modes coexist at any given frequency. Since CLamb waves have two coupled displacements (radial displacement and circumferential displacement), it is very difficult to excite a pure CLamb mode. Compared with CLamb waves, CSH waves have simpler dispersion equation and less mode conversion when encountering defects. Moreover, CSH waves have simpler wave structures and are less affected by the presence of fluid loads and coatings [21]. In addition, only the fundamental circumferential SH (CSH_0_) wave can propagate below the cutoff frequency of CSH_1_ mode and the displacement of CSH waves are decoupled with CLamb waves, which means that theoretically pure CSH_0_ mode can be excited over a wide frequency range. This characteristic is attractive since CSH_0_ mode is also practically non-dispersive over a wide frequency range as shown in Figure 1d. 

As CSH waves are attractive, especially CSH_0_ mode has practical importance in developing circumferential guided wave inspection system, much effort has been made to utilize CSH_0_ wave to detect corrosion-like defects. In laboratory studies, SH modes in plates are often used as an approximation of CSH waves in pipes [22], since numerical model and experiments in the plate are much simpler. The interaction of SH_0_ wave with wall thinning defects is highly influenced by the thinning geometry and the operating frequency range [23]. When the operating frequency is below the first cutoff frequency, the SH_0_ wave will not convert to higher order SH modes when encountering defects, so the reflection and transmission behaviors of SH_0_ wave across thinning defects are relatively simple. Demma et al. [24] investigated the effect of a rectangular notch on the propagation of the SH_0_ mode, and it was found that the reflection coefficient is strongly influenced by the axial extent (in the propagating direction) of a notch. It has minima when the axial extent is integer multiples of λ/2 and maxima when the axial extent is odd multiples of λ/4, where λ is the wavelength of SH_0_ wave. This phenomenon was also confirmed by using the principle of reciprocity of elastodynamics [25,26]. Zhao and Rose [27] used boundary element methods to analyze the SH_0_ wave scattering from a surface half-elliptical shaped defect, which is a better approximation to the corrosion defect than a notch. It was found for a low frequency incident SH_0_ wave, the reflection and transmission factors change monotonously with the defect depth. Wang et al. [28] investigated the effect of a notch on the propagation of the CSH_0_ mode in a pipe by using 3D finite element simulations. Their simulations show that the reflection and transmission coefficients of CSH_0_ waves vary monotonously with an increase in the notch depth. The above results show that CSH_0_ wave has great potential for corrosion defect sizing when its operating frequency is below the first cutoff frequency. However, there is less experimental evidence to support the CSH_0_ wave’s defect sizing capability. Even for a simple rectangular notch defect, there is no available guided-wave-based method to determine the depth of a notch. It should be noted that using SH_0_ wave to model the CSH_0_ mode in a thin pipe is accurate only for the frequency range below the first cutoff frequency. The simulations conducted by Luo et al. [22] show that, when the operating frequency is above the CSH_1_ wave cutoff frequency, the CSH_0_ wave’s reflection and transmission characteristics from a notch in a pipe are quite different from those of SH_0_ wave from a notch in a plate, even for a thin pipe with a low thickness-to-diameter ratio. Therefore, although there are some investigations which focus on the interaction of SH_0_ wave with corrosion-like defects in the high frequency-thickness regime [14,23,29,30,31], these conclusions obtained from SH_0_ wave in the high frequency-thickness regime cannot be extended to CSH_0_ wave in the pipe straightforwardly.

Undoubtedly, it is necessary to experimentally study the interaction of CSH_0_ wave with corrosion-like defects in pipes directly. However, currently, there are fewer experimental investigations due to the difficulty of exciting pure CSH_0_ (or CSH_1_) mode. To develop a CSH-wave-based SHM system, transducers should be designed to meet the following requirements: (a) The transducer should be light-weight and low-power consumption and hence it can be permanently installed on the pipe. This means that electromagnetic acoustic transducers (EMATs) are not feasible due to their relatively bulk size and high-power consumption, although EMATs can excite pure CSH_0_ wave [32]. (b) The transducer should be able to excite a pure CSH_0_ wave in one direction around the circumference of a pipe [16]. This requires that all the axial guided waves, CLamb waves, and high order CSH modes in the pipe must be suppressed by the designed transducer. In recent years, a series of face-shear piezoelectric transducers for SHM have been developed to excite single mode SH_0_ wave [33,34,35,36]. Since pure SH_0_ wave excited by the face-shear transducer is along four main directions (0°, 90°, 180° and 270°) [35], it can be inferred that if these face-shear transducers are straightforwardly used to excite CSH_0_ wave, flexural waves will be excited simultaneously in the axial direction of the pipe [11,37]. Theoretically, our recently developed bidirectional SH wave piezoelectric transducer (BSH-PT) is capable of exciting pure CSH_0_ wave in the pipe [38], but considering that there are many axial guided waves in the pipe, its capability of suppressing axial guided waves needs to be checked. (c) Besides the above two requirements, it is also expected that this transducer can operate over a wide frequency range, which means that the transducer has tunable detection sensitivity for defects of different sizes. As EMATs are usually able to excite pure CSH_0_ wave with fixed wavelength, they cannot meet this requirement. 

In this work, a piezoelectric transducer system is proposed to excite pure CSH_0_ wave and single CSH_1_ mode in large-diameter pipes and the CSH_0_ wave’s ability of sizing and monitoring corrosion-like defects in a pipe is demonstrated. Firstly, a method based on our previously developed bidirectional SH wave piezoelectric transducers was proposed to excite pure CSH_0_ wave and single CSH_1_ mode. Then, experiments were performed to explore the bidirectional transducer’s performance on exciting and receiving CSH_0_ wave over a wide frequency range. After that, a method of sizing a rectangular notch defect by using CSH_0_ wave was proposed and then confirmed by experiments. Finally, experiments were conducted to investigate the reflection and transmission characteristics of CSH_0_ wave and CSH_1_ wave from notches with different depths. The results show that the depth change of a notch can be monitored by extracting the transmission coefficient of CSH_0_ wave at different incident frequencies. 

## 2. Mechanisms of Exciting CSH_0_ and CSH_1_ Waves

**Figure 3:**
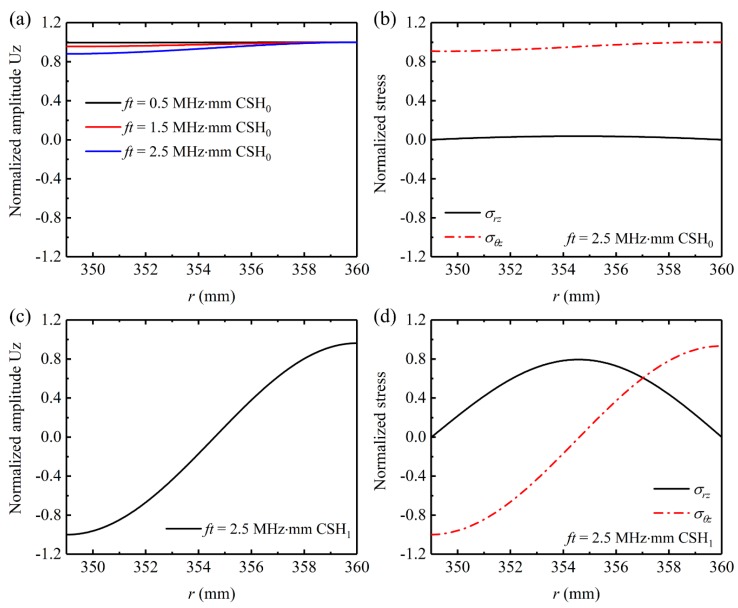
Wave structures and stresses of CSH waves in a steel pipe (outer diameter 720 mm, wall thicknesses 11 mm, Young’s modulus 210 GPa, density 7850 kg/m^3^ and Poisson’s ratio 0.33): (**a**) particle displacement distributions of CSH_0_ wave at different frequencies, (**b**) stress components $\sigma_{\theta z}$ and $\sigma_{rz}$ of CSH_0_ wave at 2.5 MHz·mm, (**c**) particle displacement distribution, (**d**) stress components of CSH_1_ wave at 2.5 MHz·mm.

**Figure 4:**
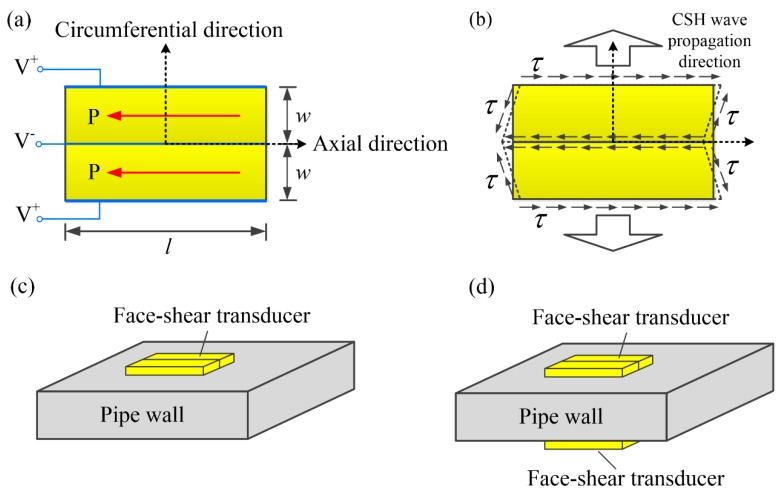
(**a**) Schematics of the proposed bidirectional CSH wave piezoelectric transducer, (**b**) bidirectional CSH wave driving mechanism, (**c**) single transducer configuration for pure CSH_0_ wave excitation below the first cutoff frequency and (**d**) dual transducer configuration for selectively exciting CSH_0_ or CSH_1_ wave above the first cutoff frequency.

The first step in developing a guided wave transducer is to analyze the wave structures in the waveguide. Figure 3a shows the particle displacement distributions of CSH_0_ wave in an 11-mm thick steel pipe at different frequencies. For better illustration, the particle displacement distributions are normalized against the maximum displacement of each case. For the CSH_0_ wave at 0.5 MHz·mm, its particle displacement is uniform across the pipe wall, which is the same as that of a plate-wave solution. Although such a uniform distribution cannot be kept when the frequency increases, the deviation is small and can be neglected especially for the cases below the first cutoff frequency. The quasi-uniform displacement distribution induces that the in-plane shear stress $\sigma_{\theta z}$ is also almost uniform across the pipe wall as shown in Figure 3b. Moreover, the shear stress $\sigma_{rz}$ of CSH_0_ wave is negligible. Therefore, the CSH_0_ wave can be generated by inducing face-shear deformation in the pipe. However, because pure SH_0_ wave in a plate is generated by the face-shear transducer along four main directions, it can be inferred that a face-shear deformation in the pipe will not only excite CSH wave but also generate axial guided waves. A previously developed bidirectional SH wave piezoelectric transducer [38] may be able to eliminate the possible guided waves propagation in the axial direction of the pipe. As shown in Figure 4a, the bidirectional transducer consists of two identical face-shear d_24_ PbZr_1−x_Ti_x_O_3_ (PZT) wafers, which are bonded together via their lateral faces. The two PZT wafers share a same electrode at the bonding interface and their polarization directions (red arrow in Figure 4a) are the same, so the induced face-shear deformations of the two PZT wafers under drive field are opposite. As demonstrated in a recent work [35], the wave field generated by a face-shear transducer is equivalent to that generated by uniform in-plane tractions along its perimeters. Since the induced shear stresses distributed along the PZT wafer edges are symmetric with respect to the axial direction, as shown in Figure 4b, it can be inferred that all the axial guided waves will be suppressed based on the symmetry principle in the theory of elastodynamics. This principle is independent of frequency, so CSH_0_ wave can be excited over a wide frequency range.

Based on the above analysis, it is known that a single bidirectional transducer configuration, shown in Figure 4c, can be used to generate pure CSH_0_ wave below the cutoff frequency of CSH_1_ mode. However, when the drive frequency is above the first cutoff frequency, the CSH_1_ mode will be also excited simultaneously by the single transducer configuration, since the in-plane shear stress $\sigma_{\theta z}$ of CSH_1_ mode is dominant on the surface of the pipe as shown in Figure 3d. Considering that the displacement and shear stress $\sigma_{\theta z}$ of CSH_1_ mode are quasi-antisymmetric across the pipe wall, as shown in Figure 3c,d, the symmetrically mounted transducer configuration shown in Figure 4d can be used to selectively excite CSH_0_ mode and CSH_1_ mode. With in-phase excitation applied on the two symmetrically-mounted bidirectional transducers, pure CSH_0_ mode can be generated while pure CSH_1_ mode can be achieved with an out-of-phase excitation [39,40].

**Figure 5:**
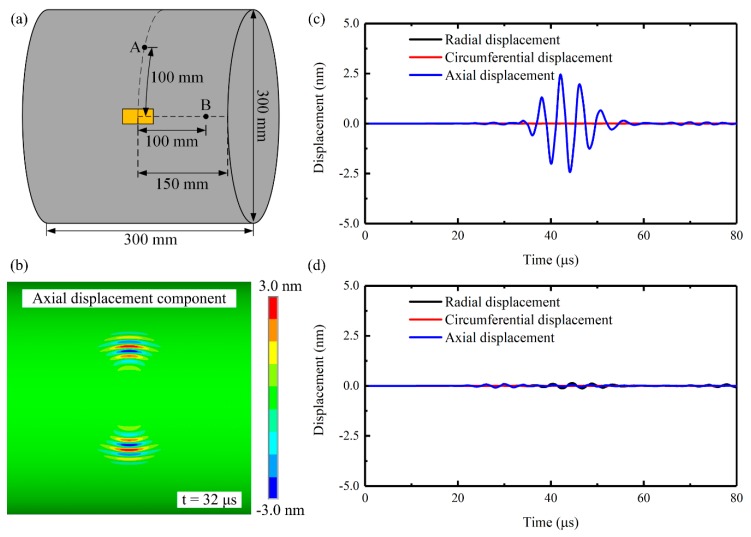
Numerical verification of pure CSH_0_ wave excitation by a single surface-bonded piezoelectric transducer at 0.25 MHz·mm in a steel pipe (outer diameter 300 mm, wall thicknesses 1 mm): (**a**) schematics of the finite element simulation setup, (**b**) axial displacement wavefield (CSH_0_ wave), displacements time history extracted from (**c**) point A and (**d**) point B.

Finite element simulations based on ANSYS software (Version 14.5 by ANSYS, Inc., Canonsburg, PA, USA) were performed to confirm the proposed excitation mechanisms. The bidirectional transducer in the simulations is made up of two rectangular PZT wafers with the dimensions of 25 mm × 6 mm × 1 mm. The material parameters of the PZT (PZT-5H) wafers can be found in the recent work [35]. Firstly, the bidirectional transducer’s performance on suppressing axial guided waves and CLamb waves was investigated. In order to reduce the computational cost, a 300 mm long, 300 mm outer diameter, and 1-mm thick steel pipe was used as the waveguide. The bidirectional transducer was bonded on the outer surface of the pipe as shown in Figure 5a. The transducer was driven at 250 kHz (corresponding to 0.25 MHz·mm in the 1-mm thick steel pipe, below the cutoff frequency of CSH_1_ mode), so by monitoring the induced displacement time history at point A and point B, the transducer’s performance on exciting pure CSH_0_ wave can be assessed. Then the thickness of the steel pipe was changed to 11 mm and two bidirectional transducers were symmetrically mounted on the outer and inner surfaces of the pipe to explore the excitation mechanism of CSH_1_ wave. The two symmetrically mounted bidirectional transducers were energized out-of-phase and the central frequency of the drive signal was 250 kHz (corresponding to 2.75 MHz·mm in the 11-mm thick steel pipe, above the cutoff frequency of CSH_1_ mode). By monitoring the induced displacement distribution across the pipe wall, the pair of bidirectional transducers’ performance on exciting CSH_1_ wave can be confirmed. In all cases, the Young’s modulus, Poisson ratio, and density of the steel pipe in the simulations were set to be 210 GPa, 0.33, and 7850 kg/m^3^, respectively. The steel pipes were modeled by SOLID185 elements, while the PZT wafers were modeled by SOLID5 elements. The largest size of elements was set to be less than 1/20 of the shortest wavelength and the time step was less than 1/(20 *f*_c_) to ensure the accuracy of the computational results, where *f*_c_ was the central frequency of the drive signal. The drive signal was a five cycle Hanning window-modulated sinusoid toneburst and its amplitude was fixed at 40 V.

Figure 5b shows the simulated axial displacement wavefield in the 1-mm thick steel pipe excited by the transducer at 0.25 MHz·mm. Based on the wave structure of CSH_0_ wave shown in Figure 3a, it is known that the axial displacement shown in Figure 5b can represent the CSH_0_ wave. As expected, the CSH_0_ wave was excited in both circumferential directions away from the transducer. To further explore the purity of the excited CSH_0_ wave, the displacements time history at point A and point B were extracted (shown in Figure 5a) and the results were plotted in Figure 5c,d. Figure 5c shows that the obtained circumferential and radial displacement components at point A are zero, indicating that no CLamb waves are generated. Moreover, Figure 5d shows that all the displacement components at point B are almost zero, which means that no axial guided waves are excited. Thus, it can be concluded that pure CSH_0_ wave was successfully excited by the bidirectional transducer.

**Figure 6:**
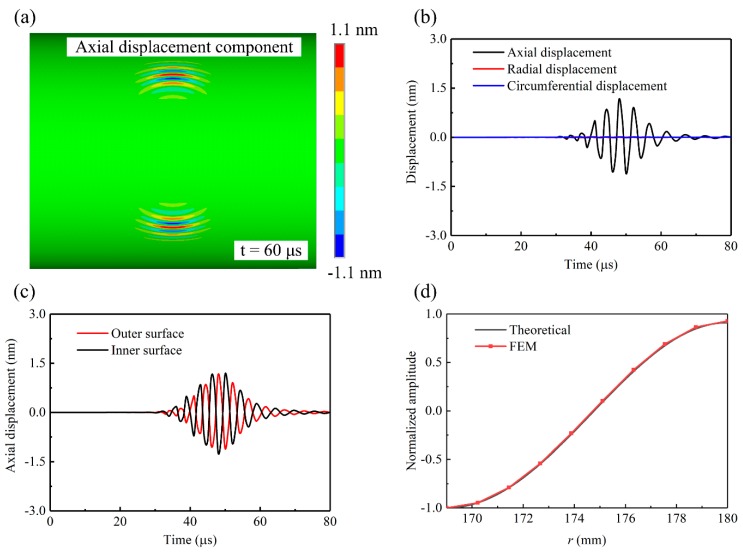
Numerical verification of pure CSH_1_ wave excitation by a pair of symmetric bonding piezoelectric transducers under out-of-phase excitation at 2.75 MHz·mm in a steel pipe (outer diameter 360 mm, wall thicknesses 11 mm): (**a**) axial displacement wavefield (CSH_1_ wave), (**b**) displacement time history extracted from the outer surface node at the circumferential distance of 30° from the transducer, (**c**) axial displacement time history extracted from the outer and inner surface nodes at the circumferential distance of 30° from the transducer and (**d**) comparison of the simulated and theoretically predicted wave structure of CSH_1_ mode.

Figure 6a presents the simulated axial displacement wavefield in an 11-mm thick steel pipe excited by a pair of symmetric bonding piezoelectric transducers under out-of-phase excitation at 2.75 MHz·mm. As expected, the wave beam was focused in two circumferential directions. The purity of the excited CSH waves can be confirmed by the displacement time history shown in Figure 6b, which was extracted from the outer surface node at the circumferential distance of 30° from the transducer. Considering that both CSH_0_ and CSH_1_ waves can propagate at 2.75 MHz·mm, the displacement time history from the inner surface node was also extracted for comparison and the results are plotted in Figure 6c. It was found that the direction of the particle vibration at the outer surface is opposite to that at the inner surface, indicating that the excited wave is CSH_1_. This can be further confirmed by the comparison of the simulated and theoretically predicted wave structures of CSH_1_ mode. Figure 6d shows that the simulated displacement distribution accords well with the theoretical wave structure of CSH_1_ mode. Obviously, the above simulation results are in good agreement with the proposed excitation mechanisms. 

## 3. Experiments 

### 3.1. Excitation and Reception of CSH_0_ Wave by the Bidirectional Piezoelectric Transducer

**Figure 7:**
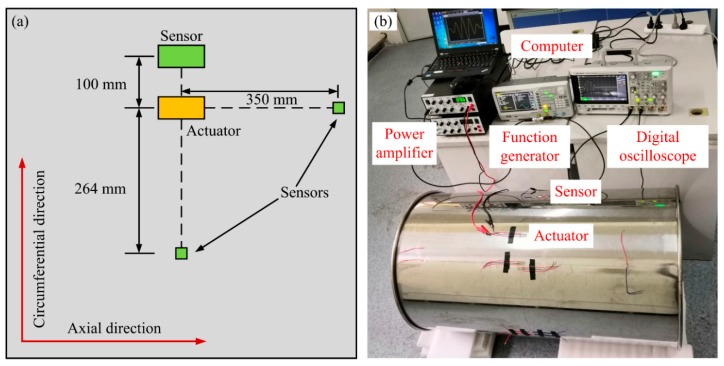
(**a**) Schematic of the experimental setup for exploring the bidirectional transducer’s performance on exciting and receiving CSH_0_ wave. (**b**) The photo of the experimental setup.

Figure 7 illustrates the experimental setup for exploring the bidirectional piezoelectric transducer’s performance on exciting and receiving CSH_0_ wave. The bidirectional transducers used in experiments have the same sizes as that used in the finite element simulations in Section 2.

As indicated in Section 2, the proposed bidirectional transducer requires that each d_24_ PZT element exhibits good face-shear performance within the operating frequency regime. Therefore, we first used a 1-mm thick steel pipe (outer diameter 600 mm, length 1000 mm) to find the frequency regime in which the designed transducer can exhibit good performance. Once the best working frequency range is determined, the transducer’s operating frequency regime in any a given-thickness pipe can be determined based on the corresponding frequency-thickness range. The bidirectional transducer for exciting CSH_0_ wave was bonded on the outer surface of the pipe using commercial epoxy resins E51 and it located at the center in the axial direction. The transducer was driven by a five-cycle Hanming window-modulated toneburst signal, which was provided by a function generator (DG4062, Rigol, Beijing, China) and amplified to 20 V by a power amplifier (KH7602M, Krohn-Hite Corporation, Brockton, MA, USA) as shown in Figure 7b. A face-shear d_36_ 0.72[Pb(Mg1/3Nb2/3)O3]-0.28[PbTiO3] (PMN-PT) single crystal wafer (d_36_ = 1600 pC/N and d_31_ = −360 pC/N, 5 mm × 5 mm × 1 mm) was firstly used as the sensor to check the purity of the excited CSH_0_ wave in the circumferential direction, since it can detect both CSH_0_ wave and the possible CLamb waves [33]. As shown in Figure 7a, the distance between the actuator and the PMN-PT sensor was set to be 264 mm. Meanwhile, another d_36_ PMN-PT sensor was placed in the axial direction to detect the possible axial guided waves. After that, another identical bidirectional piezoelectric transducer was placed in the circumferential direction 100 mm away from the actuator as the sensor to check its ability to receive CSH_0_ wave. In all cases, the signal received by sensors was collected by an oscilloscope (DSOX3024T, KEYSIGHT, Santa Rosa, CA, USA) with 128 times trace averaging. 

**Figure 8:**
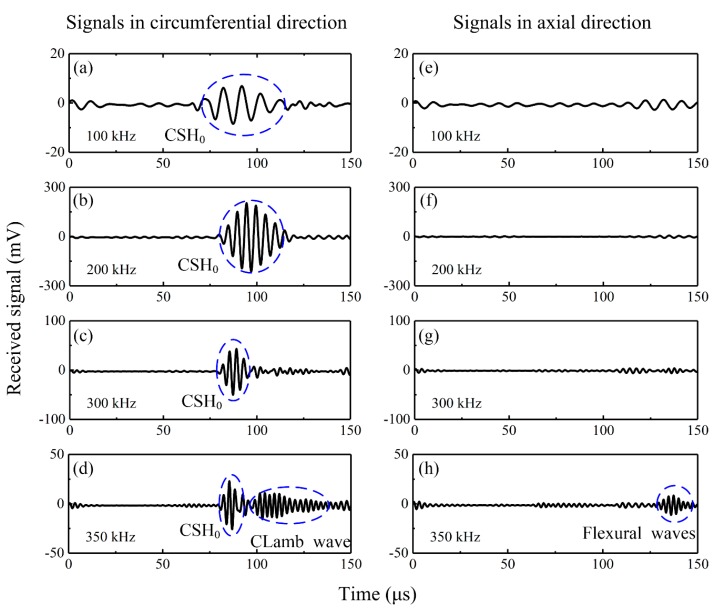
Wave signals excited by the bidirectional piezoelectric transducer at different frequencies and received by d_36_ PMN-PT sensors. (**a**–**d**) wave signals propagating in the circumferential direction, (**e**–**h**) wave signals propagating in the axial direction of the pipe.

Figure 8 presents the wave signals excited by the bidirectional piezoelectric transducer at different frequencies. Wave signals received by the d_36_ PMN-PT sensor in the circumferential direction of the pipe are plotted in Figure 8a–d. It was found that pure CSH_0_ wave was excited over a wide frequency range from 100 kHz to 300 kHz, while CLamb waves were generated at 350 kHz. Correspondingly, Figure 8e–g show that no axial guided waves were generated at the frequency range from 100 kHz to 300 kHz. When the drive frequency increased to 350 kHz, axial guided waves were also excited as shown in Figure 8h. The above phenomenon indicates that the d_24_ PZT elements used in the bidirectional transducer only exhibit good face-shear performance at the frequency range from 100 kHz to 300 kHz. At this frequency range, the bidirectional transducer can work well as the designed principle introduced in Section 2, so no CLamb waves and axial guided waves are generated. However, when the drive frequency is out of the best working frequency range, the induced deformation of the d_24_ PZT element is no longer a perfect face-shear deformation, so both CLamb waves and axial guided waves will be excited. 

**Figure 9:**
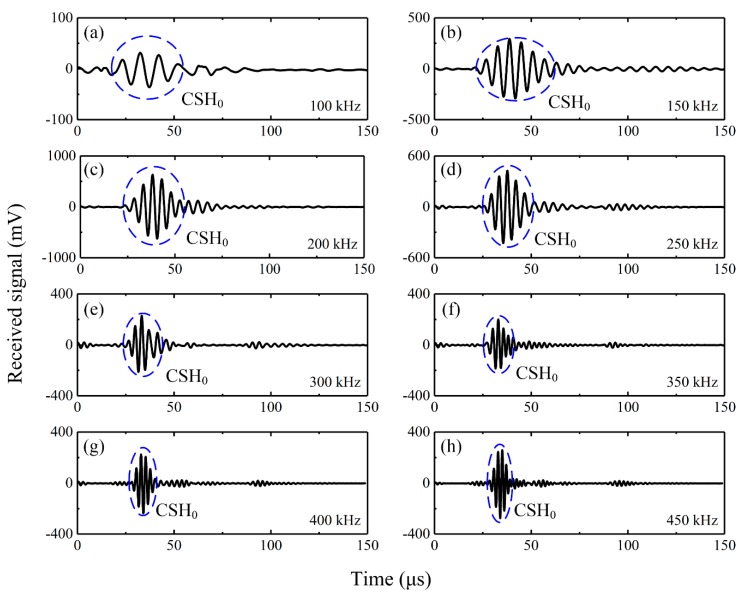
Wave signals excited by the bidirectional piezoelectric transducer at different frequencies and received by another bidirectional piezoelectric sensor in the circumferential direction of the pipe.

Figure 9 presents the wave signals generated by a bidirectional piezoelectric transducer at different frequencies and measured by another identical bidirectional transducer in the circumferential direction. It seems that pure CSH_0_ wave was excited over a wide frequency range from 100 kHz to 450 kHz. Bearing in mind that Figure 8 shows that the proposed transducer can only excite pure CSH_0_ wave from 100 kHz to 300 kHz, so Figure 8 and Figure 9 indicate that the bidirectional piezoelectric transducer can also serve as a sensor, which can receive CSH_0_ wave only by filtering CLamb waves at the frequency range from 100 kHz to 450 kHz. Obviously, the filtering capability is attractive, since it can reduce the complexity of the received signals. 

### 3.2. Sizing Corrosion-Like Defect with CSH_0_ Wave 

**Figure 10:**
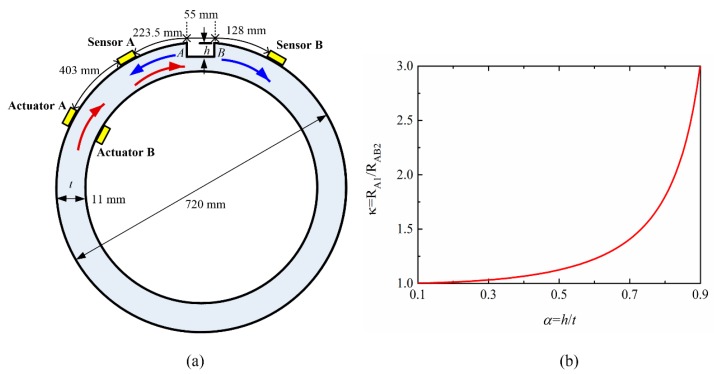
(**a**) Schematic of the experimental setup for monitoring the corrosion-like defect. (**b**) Theoretically predicted the change of the amplitude ratio κ of the two reflected signals versus the relative depth α  of the notch defect.

After confirming the proposed bidirectional transducer’s ability to excite and receive CSH waves, a CSH wave piezoelectric transducer system is constituted to monitor corrosion-like defect in thick-wall pipes. As shown in Figure 10a, an 11-mm thick steel pipe (outer diameter 720 mm, length 1000 mm) was used as the waveguide. For simplicity, a rectangular notch was used as an approximation of a corrosion type damage. Note that a real corrosion defect may have rough un-predicted surface conditions, but a rectangular notch could be a first step approximation of a corrosion type defect which is commonly used in the lab tests and theoretical studies [24,26,31]. Four identical bidirectional transducers are used to constitute the monitoring system. The layout of these transducers is shown in Figure 10a. We first consider the simple case where the operating frequency regime is below the CSH_1_ cutoff frequency. At this case, a single surface-bonded bidirectional transducer (actuator A shown in Figure 10a) is enough to excite pure CSH_0_ wave. When pure CSH_0_ wave was incident on the notch, only the CSH_0_ mode contributes to the reflection and transmission of the wave. Obviously, the CSH_0_ wave will travel back and forth inside the notch, so there will be a series of consecutive reflections. The first reflection coefficient obtained at a step down (step A shown in Figure 10a) is then given by [24]:

(1) $$R_{A1}=\frac{\alpha }{2-\alpha }$$

where α = h/t, where h is the depth of the notch and t is the original thickness of the pipe as shown in Figure 10a. The corresponding transmission coefficient past the step A would be:

(2) $$T_{A1}=\frac{2}{2-\alpha }$$

When the transmitted wave travels to the up step (step B shown in Figure 10a), part of wave energy reflects and then passes though step A, so the obtained second reflection is:

(3) $$R_{AB2}=T_{A1}\times (\frac{-\alpha }{2-\alpha })\times (\frac{2-2\alpha }{2-\alpha })=\frac{4\alpha (\alpha -1)}{{\left(2-\alpha \right)}^{3}}$$

Similarly, the amplitude of the third reflection is given by:

(4) $$R_{AB3}=T_{A1}\times {\left(\frac{-\alpha }{2-\alpha }\right)}^{3}\times (\frac{2-2\alpha }{2-\alpha })=\frac{4\alpha^{3}(\alpha -1)}{{\left(2-\alpha \right)}^{5}}$$

For a typical α = 0.5, $R_{AB3}$ is only about 3%. Therefore, the third reflection is negligible. Only the first and second reflections have practical importance for detecting the notch defect. The time delay between the first reflection and the second one is $2L/c_{g}$, where L is the circumferential extent of the notch and $c_{g}$ is the group velocity of the CSH_0_ wave. Obviously, if L is relatively large, the two reflected signals can be separated in time. This characteristic can be used to measure the circumferential extent of the notch. However, if L is too small, the reflected signals will overlap in time domain, which will induce the total reflection signal complex due to the constructive and destructive interference of the two reflected signals. The target of this work is to size the corrosion-like defect larger than 5t×5t in area and 0.5t deep where t is the original thickness of the pipe. Therefore, the circumferential extent of the notch was set to be 55 mm in the experiments as shown in Figure 10a. This extent can separate the two reflected signals in time domain, so it can be measured. The depth of the notch can be determined by using the amplitude ratio of the two reflected signals:

(5) $$\kappa =\left|\frac{R_{A1}}{R_{AB2}}\right|=\frac{{\left(2-\alpha \right)}^{2}}{4(1-\alpha )}$$

By measuring the amplitude ratio κ, the relative depth α = h/t can be obtained. It should be noted that calculation of α by using the amplitude ratio κ is better than using reflection coefficient $R_{A1}$ or $R_{AB2}$ directly, since the absolute value of the reflection coefficient is strongly influenced by the beam divergence, roughness of the pipe and the quality of bonding layer between sensor and the pipe. These factors can be minimized by using the amplitude ratio κ.

Figure 10b presents the theoretically predicted change of the amplitude ratio κ versus the relative depth of the notch defect. It can be seen that the amplitude ratio always increases with the increase of relative depth. When the relative depth α is smaller than 0.5, the ratio κ almost keeps constant, indicating that this method is insensitive to shallow defects. However, when the relative depth α becomes larger than 0.6, the increase rate of the ratio κ becomes larger and larger. Therefore, the ratio κ is a good damage index for deep defects. In order to explore the effectiveness of the proposed method, the shallowest depth of the notch in the experiments is set to be 7 mm. Then the depth of the same notch was machined in 1 mm steps to 8 mm and then to 9 mm. For each case, machining started in the shallowest depth, the measurement experiment was conducted, and then the same notch was remachined to the next depth. In all cases, the incident CSH_0_ wave was excited by the actuator A at 135 kHz and the reflected signals from the notch were received by the sensor A shown in Figure 10a. For an 11-mm thick steel pipe, 135 kHz is corresponding to 1.485 MHz·mm, which is below the cut off frequency of CSH_1_ mode, so only CSH_0_ wave can propagate.

**Figure 11:**
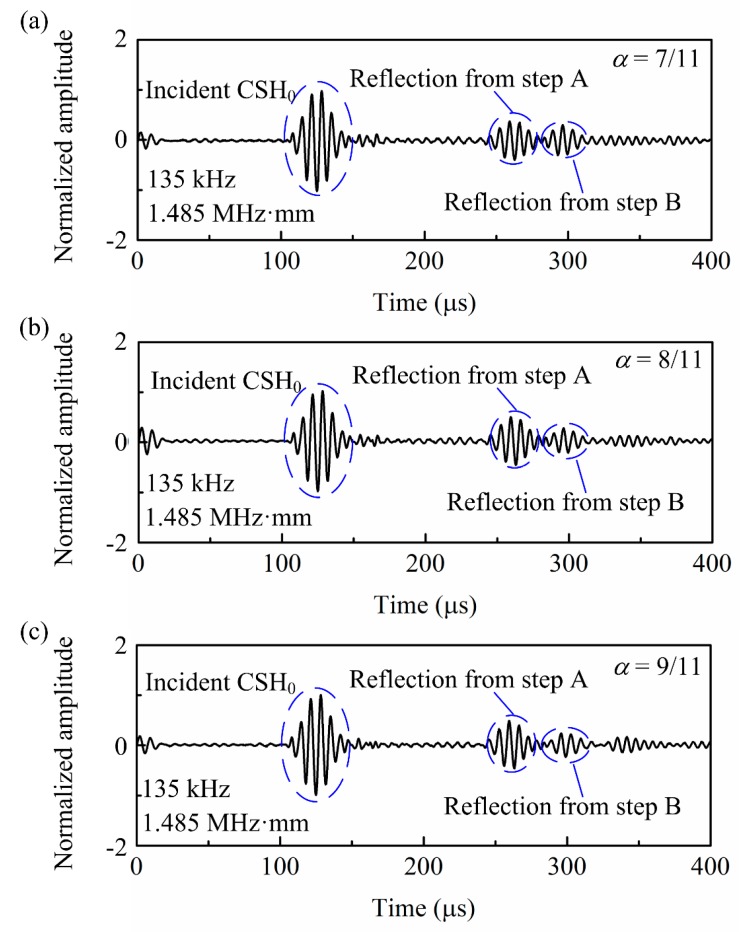
Experimental CSH_0_ wave signals showing the reflections from the rectangular notches with different depths.

Figure 11 presents the wave signals excited by actuator A at 135 kHz and received by the sensor A shown in Figure 10a. As expected, pure CSH_0_ wave was successfully generated in the 11-mm thick steel pipe. After the incident CSH_0_ wave travels across the notches, the two reflected signals can be totally separated in the time domain. By extracting the time interval of 136.15 μs between the incident signal and the reflected signal from step A, the CSH_0_ wave’s group velocity of 3283 m/s can be obtained based on the propagation distance of 447 mm shown in Figure 10a. Combining the time interval of 35.47 μs between the reflection from step A and that from step B, the circumferential extent of the notch is estimated as 58.1 mm, which agrees with the real value of 55 mm. Moreover, by extracting the amplitudes of the two reflected signals from notches with different depths, the amplitude ratio κ was calculated and then the depth of notches can be obtained from Equation (5). Table 1 presents the notch depth sizing results for the rectangular notches with different depths. As shown, the estimated depths by using the amplitude ratio κ accord very well with the true values of the notches. The estimation errors for the cases of 7 mm and 9 mm are within 2%. However, larger error is observed in the case of 8 mm, which is attributed to the machining error of the notch.

**Figure 12:**
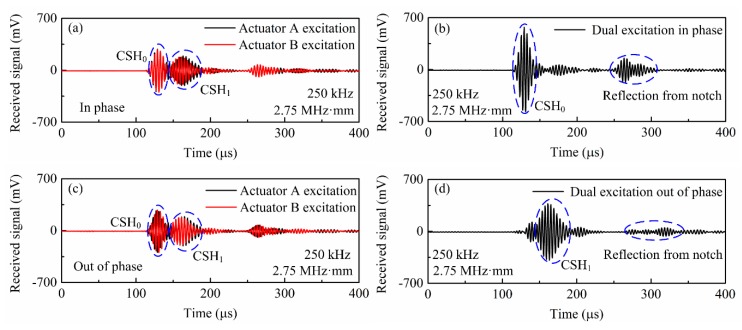
Selective generation of CSH_0_ and CSH_1_ modes by using dual excitation. (**a**) Actuator A and actuator B are driven one by one in phase, (**b**) the two transducers are simultaneously energized in phase, (**c**) the two transducers are driven one by one out-of-phase and (**d**) the two transducers are simultaneously energized out-of-phase.

Subsequently, the case where the operating frequency regime is above the CSH_1_ cutoff frequency is investigated. Note that this discussion is limited to that the pipe thickness only supports CSH_0_ and CSH_1_ modes. Figure 12a presents the wave signals generated by a single outer-surface-bonded bidirectional transducer (actuator A shown in Figure 10a) at 250 kHz and received by the sensor A. The corresponding frequency-thickness of 2.75 MHz·mm is above the cutoff frequency of CSH_1_ mode, so both CSH_0_ and CSH_1_ modes are simultaneously excited in the pipe. For comparison, the inter-surface-bonded bidirectional transducer (actuator B shown in Figure 10a) was then energized with the same drive signal used in actuator A. The obtained wave signals are also plotted in Figure 12a. As shown, the CSH_0_ wave excited by actuator A is almost the same as that generated by actuator B, while the two excited CSH_1_ modes are out-of-phase. Analogously, if the drive signal for actuator B is antiphase relative to that for actuator A, Figure 12c shows that the CSH_0_ wave excited by actuator B is also antiphase relative to that generated by actuator A, while the obtained two CSH_1_ modes are in-phase. This phenomenon is attributed that the wave structure of CSH_0_ mode is uniform across the thickness of the pipe, while the corresponding distribution of CSH_1_ mode is antisymmetric across the pipe wall as shown in Figure 3. Therefore, when actuators A and B are energized in-phase, Figure 12b shows that the excited CSH_0_ wave is significantly enhanced, while the CSH_1_ wave decreases significantly. By contrast, when actuators A and B are energized out-of-phase, Figure 12d presents that almost pure CSH_1_ mode is obtained, since the weak CSH_0_ mode can be neglected. Obviously, these results are in good agreement with the excitation mechanisms proposed in Section 2.

**Figure 13:**
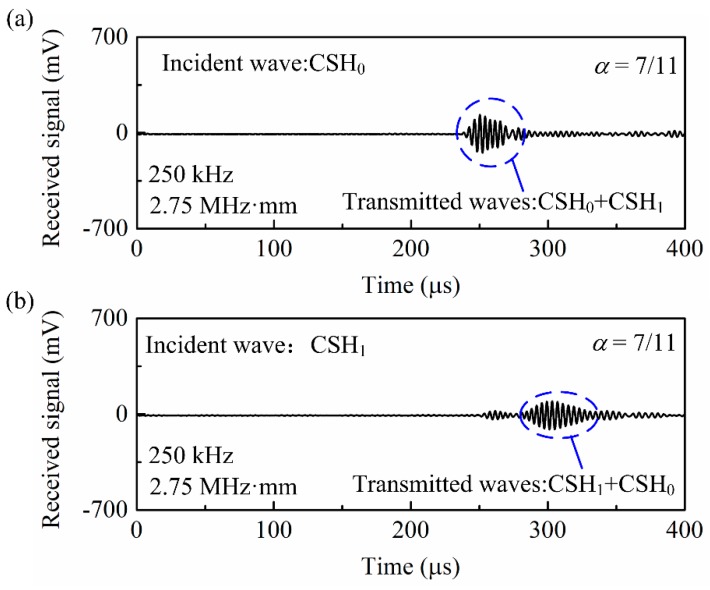
Experimental wave signals showing the transmission from a 7-mm deep rectangular notch.

Although pure incident CSH_0_ wave is generated by using dual excitation, Figure 12b shows that the reflected signals from the notch (depth: 7 mm) are still more complex than that shown in Figure 11a. The reflections contain not only the two reflected CSH_0_ waves but also the CSH_1_ mode due to mode conversion. These wave modes overlap in time domain, so the above methods to determine the depth and circumferential extents of the notch become invalid. Therefore, we move to explore the transmission signals. After the incident CSH_0_ wave passes through a 7-mm deep notch, its transmitted signals are measured by sensor B (shown in Figure 10a) and the results are plotted in Figure 13a. Obviously, both CSH_0_ and CSH_1_ modes are generated in the transmitted signals and their waveforms also overlap in the time domain. Similarly, when pure CSH_1_ mode is excited as the incident wave, the transmitted wave measured by sensor B also contains CSH_0_ and CSH_1_ modes as shown in Figure 13b.

**Figure 14:**
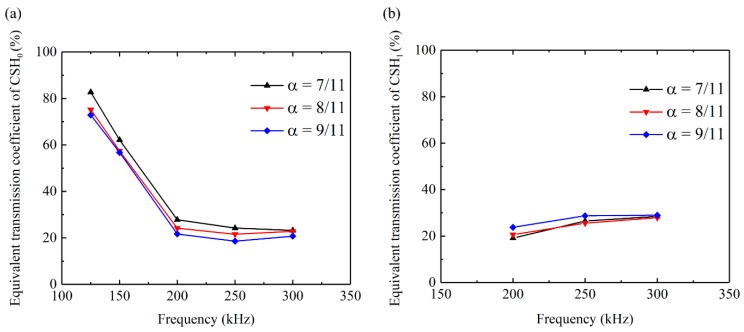
Equivalent transmission coefficients for notches with different depths by sending pure (**a**) CSH_0_ and (**b**) CSH_1_ wave at different frequencies.

By analyzing the group velocity, it is found that when the incident wave is CSH_0_ mode, most of the transmitted wave’s energy comes from the CSH_0_ mode. Analogously, when CSH_1_ mode is used as incident wave, CSH_1_ wave is the dominant mode in the transmitted signals. Therefore, by extracting the amplitude of the transmitted signal and combining the incident wave measured by sensor A, the equivalent transmission coefficient can be calculated. By sending pure CSH_0_ wave at different frequencies, the equivalent transmission coefficients for notches with different depths can be obtained as shown in Figure 14a. It can be seen that the transmission coefficients for all the notches first decrease monotonically with frequency and then keep almost constant after 200 kHz. This phenomenon accords very well with the simulations conducted by Luo, Zhao and Rose [22].

The transmission coefficient of CSH_0_ wave first decreases monotonically with frequency when the frequency range is approximately below the first cutoff frequency and then keep almost constant when the frequency range exceeds the cutoff frequency of CSH_1_ mode. This is the first experimental evidence for the simulated transmission characteristics of CSH_0_ wave across a notch at different frequencies. Moreover, Figure 14a shows that the transmission coefficients at all the frequencies decrease with increasing the notch depth. This phenomenon is valuable because it makes possible the depth monitoring through transmission coefficients. Analogously, by sending pure CSH_1_ wave at different frequencies, the corresponding equivalent transmission coefficients can also be obtained as shown in Figure 14b. It can be seen that the transmission coefficients for all the notch depths are almost independent of the frequency of the incident CSH_1_ wave. Moreover, no obvious change is observed when changing the notch’s depth. Therefore, CSH_0_ mode is more useful than CSH_1_ mode to monitor the change of the defect depth by using the transmission coefficient. 

## 4. Summary and Conclusions

In summary, a circumferential SH wave piezoelectric transducer system was developed to monitor corrosion-like defect in large-diameter pipes. Firstly, a previously proposed bidirectional SH wave piezoelectric transducer’s performance on exciting CSH waves was investigated by using both finite element simulations and experiments. Results show that the bidirectional piezoelectric transducer is capable of exciting pure CSH_0_ mode traveling in both circumferential directions of a 1-mm thick steel pipe from 100 to 300 kHz. All the axial guided waves and circumferential Lamb waves are suppressed within this frequency range. Moreover, the bidirectional transducer can also serve as a sensor to detect CSH_0_ mode only by filtering CLamb waves over a wide frequency range from 100 kHz to 450 kHz. After that, a method of sizing a rectangular notch defect by using CSH_0_ wave was proposed and then confirmed by experiments. Experiments on an 11-mm thick steel pipe show that the depth and circumferential extent of a notch can be accurately determined by using the reflection signals from the notch. This inversion method is valid only below the cutoff frequency of CSH_1_ mode. When the operating frequency is above the first cutoff frequency, dual excitation with symmetrically mounted bidirectional transducers was validated as a useful method for selectively exciting CSH_0_ or CSH_1_ mode. When pure CSH_0_ mode above the first cutoff frequency passed through notch defects, it was found that the equivalent transmission coefficients at all the frequencies decreased with the increasing of notch depth. This phenomenon was not observed when pure CSH_1_ mode was used as the incident wave. Therefore, CSH_0_ mode is more useful than CSH_1_ mode to monitor the change of the defect depth by using the transmission coefficient. This work indicates that for a simple rectangular notch, defect sizing is possible by using CSH_0_ mode. Future investigations will focus on studying the reflection and transmission characteristics of CSH waves encountering half-elliptical shaped defects, which are more approximate to corrosion defects than notches.

## Figures and Tables

**Table 1:** Notch depth sizing results for the rectangular notches with different depths.

Amplitude Ratio κ	Measured Depth (mm)	True Depth (mm)	Error
1.298	7.128	7	1.8%
1.831	8.855	8	10.7%
2.010	9.126	9	1.4%
